# MetaComb: a meta-learning framework for drug combination response prediction from cell lines to patients

**DOI:** 10.3389/fgene.2026.1811927

**Published:** 2026-06-02

**Authors:** Congcong Guo, Tongtong Li, Xinru Deng, Yajie Ma, Feng He, Lihong Diao, Ze Wang, Dong Li, Zhongyang Liu

**Affiliations:** 1 State Key Laboratory of Medical Proteomics, National Center for Protein Sciences (Beijing), Academy of Military Medical Sciences, Beijing, China; 2 Beijing Proteome Research Center, Beijing, China; 3 College of Life Sciences, Hebei University, Baoding, China; 4 College of Chemistry and Materials Science, Key Laboratory of Medicinal Chemistry and Molecular Diagnosis (Hebei University), Hebei University, Baoding, China; 5 Institute of Computing Science and Technology, Guangzhou University, Guangzhou, China

**Keywords:** drug combination, few-shot adaptation, meta-learning, precision oncology, response prediction

## Abstract

**Introduction:**

Combination therapy has emerged as a pivotal strategy in oncology to enhance efficacy and overcome drug resistance. Computational prediction models of drug combinations trained on abundant cell line data provide a starting point, but their applicability to patients remains constrained by inherent biological disparities between cultured cell lines and patient-derived tumors. However, due to ethical and cost issues, patient-derived datasets remain scarce, thus, developing patient-level predictive algorithms must explicitly confront the few-shot problem of relevant data.

**Method:**

To break through the small sample bottleneck, we used the Model-Agnostic Meta-Learning (MAML) to develop a Meta-Learning Drug Combination Response Prediction (MetaComb) method for patient *ex vivo* drug combination response prediction, using drug structures and gene expression profiles of cell lines/patients as the model input. In MetaComb, the meta-model was trained on data-rich cell line-specific drug combination response prediction tasks and subsequently fine-tuned to adapt to scenarios with limited samples.

**Results:**

MetaComb outperformed conventional transfer learning in predicting drug combination response, improving AUROC by 8.5% for data-poor cell lines and by 7.4% for patient ex vivo samples. And for the patients with *ex vivo* data, MetaComb also achieved superior accuracy over existing methods. Given the limited patient cohort, these results demonstrate the feasibility of MetaComb, but further validation with larger patient datasets is needed.

**Discussion:**

This study, as a proof-of-concept, provided an initial evidence that the MetaComb meta-learning framework is feasible for patient-derived *ex vivo* drug combination response prediction under few-shot conditions, by transferring drug-combination response knowledge from preclinical cell lines. Current patient-level assessment is insufficient to support generalization to other cancer types or patient populations, and in the future, with the accumulation of relevant patient-derived data, further validation with larger patient cohorts is required.

## 1 Introduction

Combination therapy has emerged as a crucial strategy in modern cancer treatment (Ianevski et al., 2019; Makarem and Jänne, 2024; Mc Neil and Lee, 2025). Compared with monotherapy, drug combinations can simultaneously target multiple tumor-associated signaling pathways (Jia et al., 2009), thereby improving therapeutic efficacy while reducing toxic side effects (Madani Tonekaboni et al., 2018). This strategy blocks cellular compensatory mechanisms at the molecular level, effectively addresses tumor complexity and heterogeneity, and mitigates both intrinsic and acquired drug resistance (Palmer and Sorger, 2017; Vasan et al., 2019; Wang et al., 2019)—key obstacles to achieving durable remission and improve patient outcomes. However, despite the demonstrated clinical advantages of combination therapy, identifying the optimal drug combination for individual patients remains highly challenging due to the irreversible costs of trial-and-error to patients, the exponential expansion of the combinational drug space, and the marked molecular diversity among patients.

Most existing drug combination prediction approaches have been still developed and validated within cell-line settings, however, these models are difficult to directly apply even to patient-derived *ex vivo* samples. Currently, large-scale omics data resources of cancer cell lines such as CCLE (Barretina et al., 2012), GDSC (Yang et al., 2012) and high-throughput drug combination screening databases such as DrugComb (Zheng et al., 2021) have enabled the development of numerous computational models for drug combination prediction at the cell-line level. Existing methods spanning deep neural networks (Preuer et al., 2017; Kuru et al., 2022a), graph-based models (Wang J. et al., 2022; Zhang et al., 2022), multi-task learning frameworks (El Khili et al., 2023), multimodal fusion strategies (Dong et al., 2024; Wang et al., 2025) and even meta-learning strategies (Feng et al., 2025), have substantially advanced the prediction of drug combinations in preclinical cell-line settings. Recent reviews have systematically summarized this rapidly growing field, indicating that cell-line-level drug combination prediction is already a well-established research direction (Sun et al., 2020; Wu et al., 2022). However, these preclinical cell-line efforts are difficult to directly apply even to patient-derived *ex vivo* samples. Unlike cell lines, which are immortalized, clonally selected, and adapted to *in vitro* culture, patient-derived *ex vivo* cells have a limited replicative capacity, generally retain more of the original tumor’s heterogeneity and better represent the *in vivo* state of the donor’s tissue and thus may show drug responses that are more translatable to clinical settings (Williams et al., 2022).

Owing to patient-level data scarcity, methods specifically designed for patient-level drug combination prediction remain scarce. Constrained by ethical considerations, experimental costs, and sample accessibility, currently patient-derived data are extremely limited, which hinders traditional deep learning models from adequately learning the high-dimensional molecular patterns driving drug synergy or efficacy differences (Athieniti and Spyrou, 2022; Stahlschmidt et al., 2022). Among the very limited studies in this direction, PDSP is the most closely related prior work that directly explored personalized drug synergy prediction from cell lines with rich data to patient-derived samples (Kuru et al., 2022b). PDSP first learns drug-pair synergy and single-drug sensitivity from large-scale cell-line data and then uses the patient’s a small number of *ex vivo* single-drug sensitivity measurements for model finetuning to achieve patient *ex vivo* drug combination synergy prediction tasks. Its evaluation on three leukemia patients illustrates the feasibility of transferring drug-combination prediction from cell lines to patient-derived *ex vivo* data. However, PDSP uses drug combination synergy scores as the prediction endpoint, which may not fully align with clinically meaningful outcomes, as synergy does not necessarily imply therapeutic effectiveness (Malyutina et al., 2019). Moreover, PDSP still follows a conventional pretraining-and-fine-tuning paradigm, which is not optimized for few-shot adaptation across heterogeneous tasks—a key challenge in patient-level prediction, where each patient constitutes a distinct task with extremely limited data. A traditional pre-trained model may learn source-domain biases that hinder quick adaption to patients with extremely limited data. In other words, the question of how to train a model specifically for rapid few-shot adaptation from cell lines to new patient tasks is still open.

Meta-learning offers promising solutions for rapid few-shot adaptation and has already been successfully applied in many biomedical settings such as expression profile-based single-drug response prediction (Ma et al., 2021), genomic survival analysis (Qiu et al., 2020), T-cell receptor–antigen binding recognition (Gao et al., 2023), and medical image classification (Jiang et al., 2022). One prominent approach in meta-learning is MAML (Model-Agnostic Meta-Learning), which learns an initial set of parameters that can be rapidly adapted to a new task with only a few gradient steps and a few samples. Its key advantage over many other meta-learning approaches is that it remains model-agnostic, requires no additional parameters for meta-learning, and directly optimizes for fast task-specific adaptation rather than relying on metric-based or recurrent architectures (Finn et al., 2017; Jadhav et al., 2026).

Concurrently, by representing drugs as molecular graphs and aggregating local topological information from atoms and bonds, GCN (Graph Convolutional Network) can derive high-dimensional embeddings reflecting functional and structural characteristics. Prior studies have shown the exceptional capability of GCN in capturing molecular structure and chemical semantic features and GCN-based drug representation has been successively used in many drug discovery-related algorithm constructions (Sun et al., 2020; Nguyen et al., 2022).

Motivated by these considerations, we propose MetaComb, a meta-learning framework for drug combination response prediction from cell lines to patients. Compared with existing cell-line-level drug combination prediction models, our study focuses on a more clinically relevant setting, namely adaptation from established cell lines to patient-specific *ex vivo* samples. Compared with conventional pooled learning or standard pretraining-and-finetuning strategies, MetaComb explicitly treats each cell line/patient as a task and uses MAML to learn transferable initialization parameters from data-rich cell-line tasks, thereby directly optimizing the model for rapid few-shot adaptation. This design enables adaptation not only to data-poor cell lines, but also to patient-specific *ex vivo* samples. In addition, MetaComb uses drug combination response (see Methods) as the prediction target, more closely aligned with clinically relevant efficacy than synergy alone. Our study provides initial evidence of the feasibility and effectiveness of applying meta-learning for few-shot transfer from data-rich cell line tasks to patient-derived *ex vivo* drug combination response prediction.

## 2 Materials and methods

### 2.1 Data

#### 2.1.1 Data derived from cell lines

The RNA-seq gene expression profiles for cell lines used in this study were derived from CCLE (Barretina et al., 2012). For better cross-sample comparability, RPKM (reads per kilobase of exon per million reads mapped) values of gene expression were downloaded (https://data.broadinstitute.org/ccle/CCLE_DepMap_18Q2_RNAseq_RPKM_20180502.gct) and then conversed into TPM (transcripts per kilobase million) values, using the formula:

$${TPM}_{i}=\frac{{RPKM}_{i}}{\sum_{j}RPKM_{j}}\times 10^{6}$$

Drug combination sensitivity data were sourced from DrugComb (v1.5) (Zheng et al., 2021), a publicly accessible repository compiling large-scale, high-throughput drug combination screening studies. It provides experimentally derived dose-response curves and uniformly recalculated drug combination sensitivity scores (CSS) and synergy scores (S_sum) for diverse drug combinations across diverse cancer cell lines. CSS is the average area under curve (AUC) for the drug combination’s dose-response curve with one compound fixed at the IC50 concentration and S_sum is the difference between CSS and the sum of AUCs of the monotherapy dose-response curves. When multiple CSS/S scores existed for a drug combination on a cell line (due to repeated measurements), the average value was used.

According to the criteria established by Malyutina et al. (Malyutina et al., 2019), drug combinations were defined as responsive (i.e., effective) when CSS ≥10 and S_sum >5, and as non-responsive (i.e., non-effective) otherwise. Here the CSS and S_sum scores was binarized based on the following considerations: First, clinical decision-making is inherently binary, where a patient is either a responder or a non-responder to a specific therapeutic regimen. Therefore, we converted CSS and S_sum into binary labels to align with this clinical framework. Second, binary outputs are more interpretable for clinicians, as predicting whether a patient will respond to treatment is more actionable than presenting raw CSS or S_sum scores. Lastly, binarization provides a more robust and generalizable label compared to continuous scores, which is especially important for transferring knowledge from cell lines to patient data.

**FIGURE 1:**
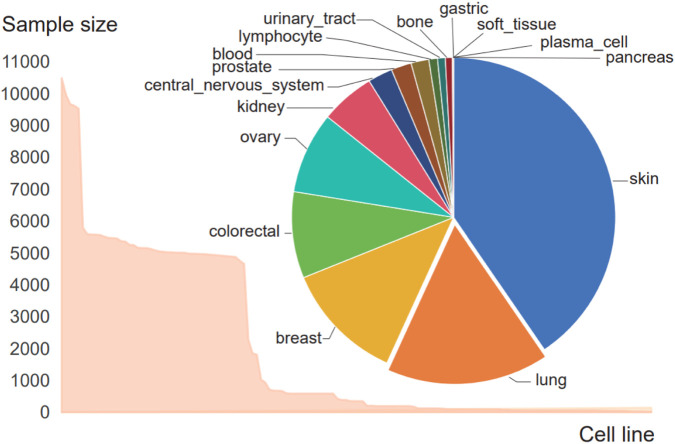
Distribution of cell line drug combination data. The distribution graph gives sample sizes (i.e., the number of drug combinations) of cell lines, with the sample size per cell line on the vertical axis and 140 cell lines in descending order of sample size on the horizontal axis. The embedded pie chart shows the distribution of all samples across various tissues.

In total, 276,087 drug combination records were compiled, covering 140 cell lines (each with ≥10 records). Descriptive statistics revealed imbalance in the distribution of known drug combination data across different cell lines (Figure 1; Supplementary Table S1). Accordingly, we classified cell lines with >50 documented drug combinations with known CSS and S_sum scores as “data-rich” cell lines (i.e., having substantial experimental data, n = 127) and those with 10–50 combinations as “data-poor” cell lines (n = 13).

#### 2.1.2 Data derived from patients

Additionally, we incorporated patient-level datasets from He et al. (He et al., 2018a), comprising transcriptomic profiles (FPKM-transformed) from three T-cell prolymphocytic leukemia (T-PLL) patients, alongside *ex vivo* screening data for 35 drug combinations tested on freshly isolated peripheral blood mononuclear cells (PBMCs) derived from patients. Similarly, FPKM values were converted into TPM values.

For each drug pair, based on drug concentrations and corresponding percentages of cell viability inhibition data from He et al., we calculated CSS and S_sum scores using the same SynergyFinder method used by the DrugComb database (He et al., 2018b; Zagidullin et al., 2019). Considering CSS and S_sum scores of cell-line level and patient-level were computed from original dose-response matrices using the same scoring pipeline, they were thought to be generally comparable, and thus we directly used the cell line-level cutoffs stated above to define patient-level drug combination response and non-response based on CSS and S_sum scores.

#### 2.1.3 Data standardization and integration

All drug compounds adopted PubChem CIDs as the standardized identifiers, with corresponding SMILES representations from PubChem (Kim et al., 2021). Genes were uniformly converted from original Ensembl IDs into Entrez Gene IDs utilizing the BioMart R package (Durinck et al., 2005), and genes with missing mappings were filtered out. An arbitrary Ensembl ID is retained when multiple Ensembl IDs correspond to one Entrez Gene ID. We identified the overlapping genes between CCLE-derived cell line expression profiles and patient expression profiles. Then for gene expression values, we further performed log2(TPM+1) conversion and gene-wise Z-score normalization. We calculated the Z-score normalization parameters (mean and standard deviation) using the training data from data-rich cell lines, and then applied these parameters to the data-poor cell lines and patient datasets to remove feature scale differences and meanwhile avoid data leakage. Ultimately, each sample was processed into a “drugA–drugB–cell line/patient” triplet, comprising two drug SMILES strings and a transcriptomic feature profile.

### 2.2 MetaComb design

**FIGURE 2:**
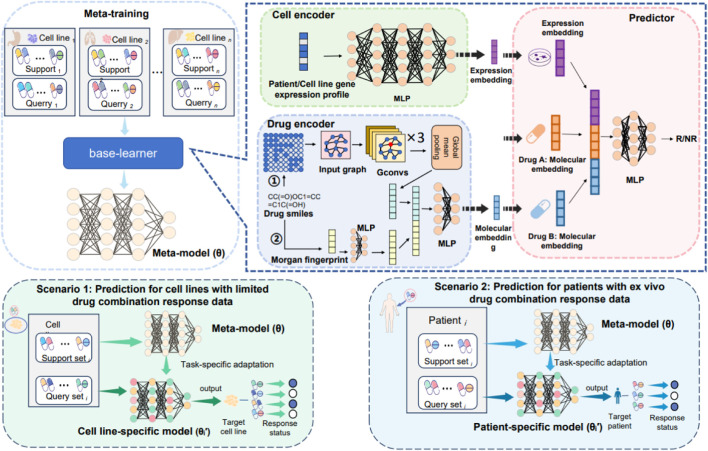
The base-learner architecture of MetaComb and two application scenarios. The base-learner comprises three modules—a drug encoder, a cell line/patient encoder, and a predictor, and it is trained on cell line–specific drug combination response prediction tasks. During meta-training, the base-learner processes task-specific data and feeds the learned information to the meta-learner; the meta-learner traverses tasks to distill cross-task meta-knowledge, enabling rapid re-parameterization and adaptation of both itself and the base-learner. The resulting meta-model is then fine-tuned with target data in two scenarios: (1) predicting drug combination responses for cell lines with scarce data, and (2) predicting drug combination responses for patients using *ex vivo* experimental data.

MetaComb implements a MAML-based algorithmic framework. MAML is a model-agnostic strategy that can be applied to any model trained with gradient descent (base-learner), optimized over a distribution of tasks. It learns an initialization that enables rapid adaptation to novel tasks, thereby achieving few-shot, data-efficient cross-task adaptation. Independent of the specific base model and task type, MAML provides a general meta-optimization paradigm that affords researchers maximal flexibility in selecting model architectures (base-learner architecture) for particular tasks, the base-learner architecture is illustrated alongside the overall MetaComb framework and application scenarios in Figure 2.

#### 2.2.1 Base-learner architecture

Firstly we built a model for expression profile-based drug combination prediction using cell line-level data merging various cell lines, as the base-learner of the subsequent meta-learning.

The base prediction model takes “drugA–drugB–cell line/patient” triplets as input instances, constructing an integrated framework with robust representation capabilities combining a dual-branch encoder and a predictor. The framework comprises three core components: (1) a drug encoder, which captures molecular structural features of drugs; (2) a cell line/patient encoder, which extracts expression profile characteristics of cells or patients; and (3) a combination prediction module, which predicts drug combination response in a given cell line or a patient. The framework is designed to simultaneously model drug molecular structures and profile features, enabling the prediction of drug combination responses in specific expression profile contexts.

##### 2.2.1.1 Drug encoder

The drug encoder is designed to learn low-dimensional feature representations from the molecular structures of drugs. Initially, SMILES strings are first parsed into molecular objects using RDKit, providing a computational representation of each drug. These molecular objects are subsequently converted into molecular graphs, where atoms are represented as nodes and chemical bonds as edges. For each atom, a 25-dimensional node feature vector is constructed, integrating the following descriptors: one-hot encoding of elemental identity (covering C, O, N, F, P, S, Cl, Br, I, H, K, Pt, and As), atomic degree, total hydrogen count, implicit valence, formal charge, binary indicators for ring membership and aromaticity, atomic mass (normalized by a factor of 100), and one-hot encoded hybridization states (including SP, SP^2^, SP^3^, SP^3^d, SP^3^d^2^). For chemical bonds, a 6-dimensional edge feature vector is generated, comprising: one-hot encoded bond types (single, double, triple, aromatic), conjugation status, and ring structural attributes. Within the graph representation, undirected bonds are explicitly modeled as bidirectional edges to preserve structural symmetry.

During the drug encoding phase, a three-layer GCN was utilized for feature aggregation across the molecular graph. The network structure is as follows:

$$Z_{1}=\text{ReLU}\left({\text{GraphConv}}_{1}\left(X_{v},A\right)\right)$$

$$Z_{2}=\text{ReLU}\left({\text{GraphConv}}_{2}\left(Z_{1},A\right)\right)$$

$$Z_{3}={\text{GraphConv}}_{3}\left(Z_{2},A\right)$$

$$E_{graph}=\text{GlobalMeanPool}\left(Z_{3}\right)$$

where $X_{v}$ represents the initial node features, A denotes the adjacency matrix, $Z_{i}$ denotes the node feature representation obtained after the i -th convolutional layer, and $E_{graph}$ is defined as the compound molecule-level vector representation, obtained by applying global mean pooling to the node representations after graph convolution.

Simultaneously, we computed Morgan fingerprints (RDKit’s GetMorganGenerator, radius = 2, dimension = 64) as global molecular descriptor $E_{morgan}$ , which was then mapped to the same dimension as the graph embedding $E_{graph}$ through a separate multilayer perceptron (MLP) to obtain the Morgan fingerprint representation ${\;E}_{desc}$ :

$$E_{desc}={\text{MLP}}_{fp}\left(E_{morgan}\right)$$

These two representations ( $E_{graph}$ and $E_{desc}$ ) were concatenated and fed into a two-layer MLP for feature fusion, yielding the final drug feature representation $E_{drug}$ :

$$E_{drug}={\text{MLP}}_{drug}\left(\text{Concat}\left(E_{graph},{\;E}_{desc}\right)\right)$$

This encoder architecture enables simultaneous capture of both local structural (atom-bond level) and global chemical property (molecular descriptor) information.

##### 2.2.1.2 Cell line/patient encoder

The cell line/patient encoder is designed to transform gene expression profiles into low-dimensional cellular representations. Its input is the high-dimensional gene expression matrix Exp (comprising 14,890 gene features) for each cell line. The network architecture consists of a five-layer MLP, structured as follows:

$$H_{1}=\text{Dropout}\left(\text{ReLU}\left(\text{LayerNorm}\left(W_{1}\left(\mathrm{Exp}\right)+b_{1}\;\right)\;\right)\;\right)$$

$$H_{2}=\text{Dropout}\left(\text{ReL}U\left(\text{LayerNorm}\left(W_{2}\left(H_{1}\right)+b_{2}\;\right)\;\right)\;\right)$$

$$H_{3}=\text{Dropout}\left(\text{ReLU}\left(\text{LayerNorm}\left(W_{3}\left(H_{2}\right)+b_{3}\;\right)\;\right)\;\right)$$

$$H_{4}=\text{Dropout}\left(\text{ReLU}\left(\text{LayerNorm}\left(W_{4}\left(H_{3}\right)+b_{4}\;\right)\;\right)\;\right)$$

$$E_{cell\;line/patient\;}={\;W}_{5}\left(H_{4}\right)+b_{5}$$

Where Exp denotes the initial gene expression features, $H_{i}$ represents the output vector of the i -th hidden layer, and $E_{cell\;line/patient}$ is the embedding vector. Except for the last layer, dropout ( p = 0.3) is applied after each layer. The output layer produces a 64-dimensional latent representation, $E_{cell\;line/patient}$ , which is subsequently fused with drug-combination features for response prediction. This architecture effectively compresses high-dimensional transcriptomic features while preserving critical expression pattern information.

##### 2.2.1.3 Combination prediction module

Upon obtaining the feature representations of both drugs and the cell line/patient transcriptomic profiles, the model concatenates these three components into a vector $E_{all}$ :

$$E_{all\;}=\text{Concat}\left(E_{drug1},E_{drug2},E_{cell\;line/patient}\right)$$

The joint representation is then processed by a three-layer MLP for feature fusion and nonlinear transformation, ultimately producing predicted response probabilities through a Sigmoid activation function:

$$\hat{y}=\text{Sigmoid}\left({\text{MLP}}_{pred}\left(E_{all}\right)\right)$$

#### 2.2.2 MAML

MetaComb used the MAML framework to achieve few-shot learning, which was divided into meta-training and meta-testing two phases. The previously constructed base-learner for prediction was incorporated into the MAML framework, where cell line-specific drug combination response prediction tasks served as the meta-training tasks to train the meta-learner. The meta-model was optimized to learn transferable “drug pair–gene expression” patterns. Transfer scenarios were progressively introduced for meta-testing, and few-shot fine-tuning was applied to enable adaptation to novel contexts.

##### 2.2.2.1 Meta-training

We divided the data-rich cell lines into 127 tasks according to the cell line type, then each task was divided into a support set (S) and a query set (Q). For a task $T_{i\;}$ (corresponding to a sample set from a specific cell line type), we randomly selected 50 samples with equal number of positive and negative ones and divided them evenly into the support set $S_{i}$ and the query set $Q_{i}$ . Using these cell line-specific drug combination prediction tasks for meta-training, we aimed to learn a general meta-model (i.e., an optimal parameter vector θ ) capable of rapidly adapting to novel tasks through few-shot fine-tuning.

The meta-training procedure consists of inner-loop and outer-loop updates. Within each meta-training cycle, the model performs parameter updates through batches of multiple tasks. The inner-loop focuses on task-specific adaptation: using the provided support set data, it executes few-step gradient updates on the base-learner’s parameters to optimize within-task loss; while the outer-loop centers on cross-task generalizable learning: it aggregates query-set losses from the model adapted to multiple tasks, updating the shared initialization parameters, ultimately obtaining the optimal parameters for the meta-model.

Within the meta-learning framework, the overall optimization objective comprises both support set loss $L_{S}$ and query set loss $L_{Q}$ , aimed at achieving both rapid task-specific adaptation and optimization of shared knowledge across tasks. The meta-objective function can be expressed as:

$$L_{meta}=E_{T_{i}∼p\left(T\right)}\left[L_{Q_{i}}\left(\theta_{i}^{\prime }\;;T_{i}\right)\right]$$

which means that it minimizes the expected loss over the query sets across all tasks $T_{i}$ sampled from the task distribution $p\left(T\right)$ .

For each task $T_{i}$ , the model parameters θ are first updated in the inner loop using the $S_{i}$ , yielding task-specific parameters $\theta_{i}^{\prime }$ , with the update rule given by:

$$\theta_{i}^{\prime }=\theta -\alpha \nabla_{\theta }L_{S_{i}}\left(\theta \;;T_{i}\right)$$

where α denotes the learning rate for the inner-loop updates, $L_{S_{i}}$ represents the loss obtained from $S_{i}$ .

Subsequently, the loss $L_{Q}$ is computed on the query set, and the shared initialization parameters θ are optimized through the outer-loop updates, thereby enhancing the model’s generalization capability on unseen tasks. The optimization objective for the outer loop is given by:

$$\theta \leftarrow \theta -\beta \nabla_{\theta }\sum_{T_{i}∼P\left(T\right)}L_{Q_{i}}\left(\theta_{i}^{\prime }\;;T_{i}\right)$$

where $\sum_{T_{i}∼P\left(T\right)}L_{Q_{i}}\left(\theta_{i}^{\prime }\;;T_{i}\right)$ represents the total loss of all tasks on their respective query sets $Q_{i}$ , and β represents the learning rate of the outer loop.

For meta-optimization, the Adam optimizer was employed with an outer-loop learning rate of 3 × 10^−4^ and an inner-loop learning rate of 0.1. An early stopping mechanism (patience = 20) was incorporated during model training. This mechanism would terminate training if the model loss failed to decrease for 20 consecutive epochs, effectively mitigating overfitting while preserving the best-performing model in each run for subsequent evaluation.

The meta-training strategy enables the model to learn universal representation initialization from source-domain cell lines while achieving rapid transfer and adaptation to target-domain tasks.

##### 2.2.2.2 Meta-testing

MAML equips the meta-model with rapid adaptation capability using few samples. We assessed model generalization performance in two scenarios: data-poor cell line transfer and patient transfer.

We first evaluated model generalization through cell line transfer tasks. As previously described, cell lines were categorized into “data-rich” cell lines and “data-poor” cell lines based on sample size. Meta-training was performed on data-rich cell lines (source domain data) to derive a universal meta-model. For “data-poor” target cell lines, they were similarly divided into distinct tasks according to cell line type, with samples evenly distributed between support and query sets. The support set fine-tuned the trained meta-model to obtain the cell line-specific drug combination prediction model, while the query set evaluated model performance.

In scenario 2, near-clinical transfer evaluation was performed for the transfer from cell line to *ex vivo* drug combination response in real patient peripheral blood samples: Target tasks were constructed from three clinical T-cell prolymphocytic leukemia (T-PLL) patient samples reported by He et al. (He et al., 2018a). For each individual patient, their *ex vivo* drug combination data were equally divided into support and query sets. First, three rounds of rapid fine-tuning were conducted using each patient’s support set to build personalized drug combination response prediction models. Subsequently, prediction performance of these individualized models was evaluated on the corresponding query sets from the same patients. Response definitions (e.g., synergy scores) strictly followed CSS/S_sum criteria to ensure consistency with the evaluation standards used for cell line tasks.

#### 2.2.3 Loss function

The model employed the binary cross-entropy (BCE) loss function to quantify the discrepancy between predicted drug combination response probabilities and ground truth labels.

For a fundamental sample unit “*drugA–drugB–cell line/patient*” triplet as mentioned above, we simply represent them as the triplet notation “ $d_{1}-d_{2}-c/p$ ”.

For a given sample consisting of drug pair $d_{1}-d_{2}$ tested on cell line c or patient p , the model outputs a predicted response probability ${\hat{y}}_{\left(d_{1},{\;d}_{2},c/p\right)}\in \left[0,1\right]$ , indicating the likelihood of therapeutic response to the drug combination in the specific cellular or patient context. The experimentally derived ground truth label $y_{\left(d_{1},d_{2},c/p\right)}\in \left\{0,1\right.$ } assigns 1 for response and 0 for non-response. The loss function is defined as follows:

$$L_{BCE}=-\frac{1}{N}\sum_{\left(d_{1},d_{2},c/p\right)\in D}\left[y_{\left(d_{1},d_{2},c/p\right)}\log\left({\hat{y}}_{\left(d_{1},d_{2},c/p\right)}\right)+\left(1-y_{\left(d_{1},d_{2},c/p\right)}\right)\log\left(1-{\hat{y}}_{\left(d_{1},d_{2},c/p\right)}\right)\right]$$

where N denotes the number of samples, and D represents the dataset for the current task.

#### 2.2.4 Performance evaluation

Performance was assessed using area under the receiver operating curve (AUROC), area under the precision-recall curve (AUPRC), which are widely used for the performance evaluation of the classification task. All reported results were averaged over multiple runs, to minimize stochastic perturbations from model initialization and data splits.

### 2.3 Model comparison

To enable quantitative evaluation of the meta-learning framework’s superiority in few-shot learning, we established two controls: 1) a baseline model without transfer learning, and 2) a conventional transfer learning model (non-meta-learning framework).

The baseline model was trained based on the mixed data from data-rich cell lines (i.e. meta-training data) and the fine-tuning sets of all target tasks. The transfer learning model was first pretrained using the mixed data from data-rich cell lines and subsequently fine-tuned with the fine-tuning set of each target task.

The “baseline model” was trained using all meta-training data (source domain data) alongside fine-tuning sets from all target tasks (partial target domain data), while the “transfer learning model” was first pretrained using data-rich cell lines data (source data) and subsequently fine-tuned using target-task-specific datasets (partial target data). To ensure a fair comparison, all control models were strictly consistent with MetaComb across key dimensions including the basal model architecture, training and testing data, and replicate experiments.

## 3 Results

### 3.1 The performance assessment of the base-learner

As the MAML framework is model-agnostic, we first established a base-learner model (Methods; Figure 2) serving as the foundation model of subsequent MAML meta-learning. Most existing drug cell-level models use drug combination synergy/antagonism as the prediction objective (Baptista et al., 2022; Wang J. et al., 2022; Wang X. et al., 2022; Li et al., 2023). However, synergy observed in a cell line does not necessarily imply therapeutic effectiveness, and even highly synergistic combinations may fail to kill cancer cells efficiently (Duarte and Vale, 2022; Jaaks et al., 2022; Vis et al., 2024). Our ultimate goal is to transfer the meta-model trained on cell-line tasks to patient-level drug combination response prediction, so it is essential to prioritize drug combination response rather than synergy at the cell-line level. Therefore, here we used drug combination response—quantified by CSS and S_sum scores (see Methods)—as the prediction target at the cell-line level, which more closely aligns with patient “response” (Jaaks et al., 2022; Vis et al., 2024).

**FIGURE 3:**
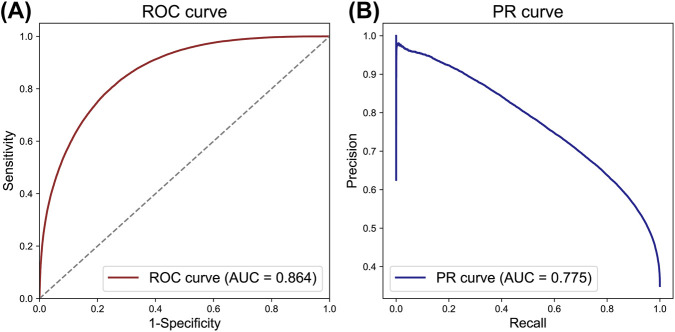
Performance of MetaComb’s base-learner. The **(A)** ROC and **(B)** PR curves and their area under curves(AUCs) of the base learner using the mixed data of data-rich cell lines as the golden standard dataset and based on 5-fold cross-validation.

Using the mixed data of data-rich cell lines as the gold-standard dataset, we assessed the performance of the base-learner, 5-fold cross-validation demonstrated robust performance (AUROC = 0.86, AUPRC = 0.78) (Figure 3). These results confirmed the base-learner’s effectiveness in the task of drug combination response prediction, validating its suitability as a base architecture for subsequent meta-learning.

### 3.2 Drug combination response prediction for cell lines with poor data

Before applying MetaComb to adapt patient-level prediction tasks, we first tested its few-shot adaption ability on data-poor cell lines, laying foundation for ultimate patient-level adaption and performance evaluation.

Drug combination data on cell lines is crucial for the development of combination therapies. However, the existing data on drug combinations across cell lines exhibit significant imbalance, primarily attributable to heterogeneity in sampling feasibility and disparities in cancer type incidence rates. For instance, cell lines originating from tissues such as skin have extensive data, while there is a lack of relevant data in cell lines from pancreas (Figure 1). The need for predicting drug combinations on cell lines with limited experimental data is more urgent and poses greater challenges compared to those with rich data. Here we assessed MetaComb’s adaption ability for drug combination prediction on data-poor cell lines.

For this scenario, using the model constructed before (see Methods, Section 2.1.) as the base-learner structure, MAML framework was adopted to train the meta-model with data-rich cell lines. Then for a cell line with poor data, the cell line specific model was constructed by fine-tuning the meta-model with a small number of samples. We compared MetaComb with the baseline model and conventional transfer learning model (see Methods).

**FIGURE 4:**
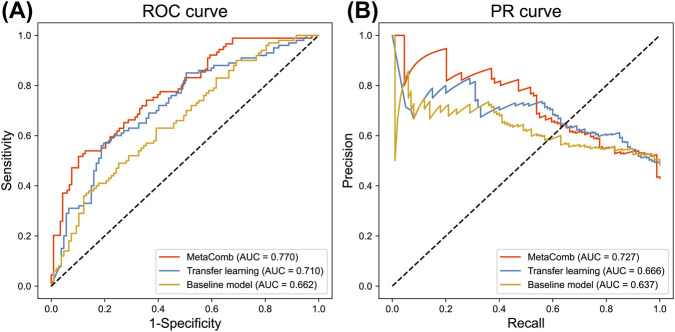
The **(A)** ROC and **(B)** PR curves and their AUCs of MetaComb and two control models for scenario 1: drug combination response prediction for cell lines with poor data. Here, the “Transfer learning model” was trained based on the mixed data of data-rich cell lines and fine-tuned with the fine-tuning set of target tasks, the “baseline model” was trained based on the mixed data from data-rich cell lines and the fine-tuning sets of target tasks. For transfer learning model and MetaComb, prediction results of the test sets of all poor-data cell lines were merged to draw the ROC/PR curve. The reported AUROCs and AUPRCs were averaged over multiple runs and the ROC and PR curves were plotted based on a single representative run.

In result, we saw that (Figure 4), MetaComb achieved apparently better prediction performance (AUROC = 0.77) than the baseline model (AUROC = 0.66), and the conventional transfer learning model (AUROC = 0.71). This result indicated the superiority of the MAML framework on the drug combination response prediction for cell lines with poor data. We inferred that the conventional transfer learning model (trained on merged cell line data) was less effective than MAML-based MetaComb at capturing shared knowledge across cell lines, and thus its ability of rapid adaption to new cell lines was not as good as that of MAML. In addition, the better performance of the 2 cell line-specific models (MetaComb and the transfer learning model) also indicated that for cell lines with poor data, the construction of cell-line specific model is necessary. The non-cell line specific meta-model trained solely on data-rich cell lines ignores data imbalance, leading to inaccurate predictions for those with sparse data.

### 3.3 Drug combination response prediction for patients with *ex vivo* experimental data

Here, we applied MetaComb to a near-clinical scenario, i.e. drug combination response prediction for three patients from He et al.'s study (He et al., 2018a). For these 3 T-PLL patients, He et al. performed drug combination testing on fresh peripheral blood mononuclear cell samples (80% leukemic cells), involving 35 drug combinations. Each patient corresponded to a prediction task, which we considered as a few-shot learning problem. We used part of the data of a patient to fine-tune the meta-model (trained based on data-rich cell lines with the help of MAML) to build the patient-specific model, and used the remaining data to assess the model’s prediction performance.

**FIGURE 5:**
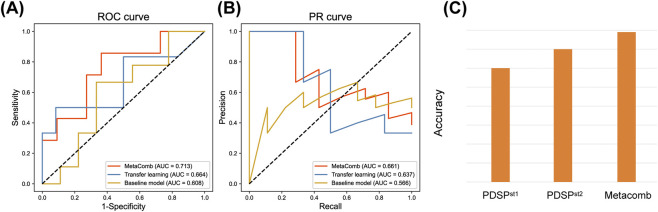
Patient-specific prediction model’s performance. **(A,B)** The ROC and PR curves and their AUCs of MetaComb and two control models for drug combination response prediction for patient *ex vivo* samples. Here, the transfer learning model was trained on the mixed data from data-rich cell lines and subsequently fine-tuned on the fine-tuning set of target patient tasks; the baseline model was trained directly on the mixed data from data-rich cell lines and the fine-tuning sets of target patient tasks. Prediction results of the test sets of all patients were merged to draw the ROC/PR curves. The reported AUROCs and AUPRCs are averaged over multiple runs and the ROC and PR curves were drawn using a single run. **(C)** Prediction accuracy of MetaComb, PDSP^st1^ and PDSP^st2^. Here the accuracy for MetaComb was the mean of 30 repetitions, while the accuracy scores of PDSPst1 and PDSPst2 were taken from Kuru et al.'s paper (He et al).

Based on identical fine-tuning and test sets, we compared the prediction performance of MetaComb with the baseline model and conventional transfer learning model, the construction details of which were similar to scenario 1 (see Methods, Section 2.5). Evaluation showed that the AUROC of MetaComb was 0.71 (Figure 5A), and the AUPRC was 0.66 (Figure 5B), apparently better than the two control models, indicating the feasibility of MetaComb for patient *ex vivo* drug combination response prediction with limited data.

Further, we compared the performance of MetaComb with PDSP, which, is the most directly relevant prior method as stated in Introduction (Kuru et al., 2022b). PDSP first pre-trained a cell line-level model (using data merging diverse cell lines) similarly using chemical structures of drugs and gene expression profiles of cell lines as the input feature but using both the single-drug sensitivity and dual-drug synergism as the prediction targets. Then, Kuru et al. used patient single-drug sensitivity data to fine-tune the model, and used the tuned model for patient drug combination synergy prediction. PDSP^st1^ and PDSP^st2^ were different in fine-tuning strategies. The former fine-tuned the model using all the three patients’ single-drug response data, while the latter used each patient’s single-drug response data separately.

Because PDSP used drug combination synergy binarization (a positive Bliss score is considered as synergy, and a negative score antagonism.) rather than drug response binarization as the prediction target, for fairness, here, the comparison scheme is: 1) we also used the same drug combination synergy binarization as the prediction target to re-train MetaComb and 2) further we used PDSP’s test samples to assess MetaComb and compared the prediction accuracy (ACC) of MetaComb with those of PDSP^st1^ and PDSP^st2^ reported in their paper. The prediction results of all patient samples were merged to compute the prediction accuracy. In result, the accuracy of MetaComb was apparently higher than that of PDSP^st1^ and PDSP^st2^ (ACC = 0.79, Figure 5C). The result further indicated the feasibility of meta-learning framework and its superiority over traditional pretraining-finetuning models on rapid adaption to limited data conditions.

## 4 Discussion

Combination therapy is pivotal for overcoming tumor heterogeneity and drug resistance, yet patient-level drug combination prediction is severely hindered by the scarcity of labeled patient-level data. MetaComb introduces a meta-learning framework that explicitly addresses the data scarcity bottleneck. MetaComb outperforms baseline and transfer learning models in predicting drug combination response for data-poor cell lines and especially for patient *ex vivo* samples. This superiority highlights the quick adaption ability of MetaComb in few-shot conditions.

In this study, we directly used the cutoffs on the cell line to define patient *ex vivo* drug combination response/non-response from CSS and S_sum scores, mainly because CSS and S_sum scores of both sides are generally comparable through a consistent scoring pipeline as mentioned in Methods. However, we acknowledge that the cutoffs are only operational thresholds in patient context. They have not been clinically validated and their applicability to real-world patient samples remains uncertain. Therefore, we have added supplementary analyses to examine the robustness of the results under alternative threshold settings. In result, we find that MetaComb has still achieved better patient-level *ex vivo* prediction performance than baseline and ordinary transferring learning models under alternative thresholds (Supplementary Table S2). This indicates the robustness of the conclusion to thresholds. Moreover, in this study we converted drug combination efficacy and synergy scores into binary labels for response/non-response classification. It has better clinical relevance, but binarization may result in potential loss of quantitative information. Therefore, we also supplemented regression-based analyses using drug combination efficacy scores CSS as the fitting target. The additional regression results show that MetaComb still outperformed the baseline model and the conventional transfer learning model for poor-data cell lines and especially for patient *ex vivo* samples (Supplementary Table S3; Supplementary Table S4), supporting the robustness of our conclusions beyond the binary classification setting.

The main objective of this work is not to establish a new method for conventional cell-line task, but to evaluate whether meta-learning improves few-shot adaptation from cell lines to patient *ex vivo* settings with limited samples. For the main objective, in this study, we chose a non-transfer baseline and a conventional transfer-learning model as the benchmark, so that the additional contribution of the meta-learning framework itself to the patient-level adaption could be assessed more clearly. In addition, we compared MetaComb with PDSP, which, to the best of our knowledge, is the published framework that is the most directly relevant to our primary objective (personalized drug-combination prediction from cell lines to patients). Data-poor cell line scenario is mainly aimed at accumulating modeling experience and laying the foundation for the ultimate patient scenario. Broader benchmarking against recent high-performance cell-line-level models would be also very valuable, but such comparisons would require careful adaptation to the same transfer setting for fairness. This will be a future direction.

The current transcriptomic data preprocessing considers cross-dataset comparability at the level of feature scaling through log2(TPM+1) transformation and source-derived gene-wise Z-score standardization. However, this doesn’t fully resolve cross-dataset comparability or batch-effect issues. Residual differences may still remain, arising from both technical variation between datasets and genuine biological differences between cell lines and patient-derived samples. However, we think that it should be very cautious about applying more aggressive batch-correction methods between cell line and patient datasets. Methods such as ComBat (Johnson et al., 2007) may reduce technical differences, but in this setting they may also remove biologically meaningful differences between cell lines and patient-derived samples. Therefore, we haven’t performed batch-effect correction. Instead, we adopted a more conservative normalization strategy and expected the few-shot transfer model to learn transferable patterns and adapt to the cross-domain differences.

In addition, in current study, patient-level assessment was done on extremely limited data (3 patients), which may introduce large variance in performance estimates and insufficient statistical power. Meanwhile the current patient cohort is insufficient for generalizability evaluation to other cancer types, disease contexts, or broader patient populations. And current patient-level evaluation is designed to assess within-patient few-shot adaptation, rather than validation of generalizability across patients. This study, as a proof-of-concept study, is intended to provide initial evidence that the proposed meta-learning framework is feasible for transfer from cell line data to patient-derived *ex vivo* settings under few-shot conditions. In future, with the accumulation of relevant available patient-derived data, performance and generalization ability (including cross-patient generalization ability) of MetaComb will be more fully explored. Further, to achieve true clinical translation, future work will also expand validation on patient *in vivo* settings. leveraging labeled clinical data from databases such as the Cancer Treatment Response gene signature DataBase (CTR-DB) (Liu et al., 2022; Jiang et al., 2025) and the Cancer Drug-induced gene expression Signature DataBase (CDS-DB) (Liu et al., 2024).

Finally, while we utilized graph and transcriptomic features, the multi-modal granularity can be deepened; incorporating single-cell spatial transcriptomics and multi-granular compound features (such as functional groups and 3D conformations) would further refine the biological resolution of our predictions. Moreover, our current approach relies on structured data, overlooking the vast unstructured domain knowledge (e.g., literature and clinical notes). Integrating pre-trained Large Language Models (LLMs) could allow us to incorporate domain priors and semantic knowledge into the prediction loop.

## 5 Conclusion

Cancer drug resistance is prevalent due to tumor heterogeneity. Drug combination is an effective strategy against drug resistance and has been widely used in cancer clinical treatment. Drug response prediction of patients is a crucial task in precision oncology, which puts forward an urgent demand for the development of bioinformatics algorithms. However, due to ethical and cost issues, the related patient data are relatively scarce, bringing challenges for the study of related prediction algorithms. To break through the small sample bottleneck, in this study we used the idea of meta-learning to develop MetaComb method. MetaComb trained the meta-model using cell line-specific drug combination efficacy prediction tasks with sufficient data, and fine-tuned it to adapt to the scenarios with small samples. MetaComb leveraged meta-learning to capture transferable drug-gene interaction patterns through drug molecular graphs and transcriptomic profiles, enabling rapid task adaptation for prediction. The assessment on three patients showed the effectiveness and superiority of the meta-learning in overcoming the small-sample bottleneck of patient *ex vivo* drug combination response prediction, providing an initial proof-of-concept for transferring preclinical drug-combination response knowledge to patient-derived *ex vivo* settings, though further validation with larger patient cohorts is required.

## Data Availability

The original contributions presented in the study are included in the article/Supplementary Material and are available at https://github.com/liuzy1984/MetaComb, further inquiries can be directed to the corresponding authors.
