# Supplementary material for: MetaComb: a meta-learning framework for drug combination response prediction from cell lines to patients
Source: Front Genet. 2026 Jun 2;17:1811927. doi: 10.3389/fgene.2026.1811927 (PMC13268602; doi:10.3389/fgene.2026.1811927)
Supplement: Supplementary file 1 [file Table2.docx]

**Supplementary Table 2** Performance assessment results of MetaComb and two control models for patient ex vivo drug combination prediction under different CSS/S_sum thresholds used to define response/non-response classification labels of patients.

| CSS cutoff=10, S_sum cutoff =5 | | |
| --- | --- | --- |
| Model | AUROC(average) | AUPRC(average) |
| MetaComb | 0.7127 | 0.6611 |
| Baseline model | 0.6075 | 0.5657 |
| Transfer learning | 0.6644 | 0.6366 |
|  |  |  |
| CSS cutoff=5, S_sum cutoff=0 | | |
| Model | AUROC(average) | AUPRC(average) |
| MetaComb | 0.6236 | 0.6857 |
| Baseline model | 0.4903 | 0.6572 |
| Transfer learning | 0.5618 | 0.6486 |
|  |  |  |
| CSS cutoff=15, S_sum cutoff=10 | | |
| Model | AUROC(average) | AUPRC(average) |
| MetaComb | 0.7930 | 0.6621 |
| Baseline model | 0.6018 | 0.5364 |
| Transfer learning | 0.6659 | 0.5094 |
|  |  |  |
| CSS cutoff=20, S_sum cutoff=20 | | |
| Model | AUROC(average) | AUPRC(average) |
| MetaComb | 0.8972 | 0.5776 |
| Baseline model | 0.7233 | 0.3508 |
| Transfer learning | 0.6484 | 0.3142 |

**Supplementary Table 3** Performance assessment results of regression-version MetaComb and two control models fitting CSS scores for data-poor cell line drug combination prediction. The Pearson correlation coefficient measures the linear relationship between predicted and true values, calculated as their covariance divided by the product of their standard deviations. It ranges from -1 (perfect negative) to 1 (perfect positive), with 0 indicating no linear correlation.The Spearman rank correlation coefficient measures the monotonic relationship between predicted and true values using their ranked values instead of raw data. It also ranges from −1 (perfect negative) to 1 (perfect positive), with 0 indicating no monotonic relationship.

| Model | Pearson(average) | Spearman(average) |
| --- | --- | --- |
| MetaComb | 0.7226 | 0.6947 |
| Baseline model | 0.6966 | 0.6837 |
| Tranfer learning | 0.7023 | 0.6701 |

**Supplementary Table 4** Performance assessment results of regression-version MetaComb and two control models fitting CSS scores for patient ex vivo drug combination prediction. The Pearson correlation coefficient measures the linear relationship between predicted and true values, calculated as their covariance divided by the product of their standard deviations. It ranges from −1 (perfect negative) to 1 (perfect positive), with 0 indicating no linear correlation.The Spearman rank correlation coefficient measures the monotonic relationship between predicted and true values using their ranked values instead of raw data. It also ranges from −1 (perfect negative) to 1 (perfect positive), with 0 indicating no monotonic relationship.

| Model | Pearson(average) | Spearman(average) |
| --- | --- | --- |
| MetaComb | 0.7416 | 0.7065 |
| Baseline model | 0.6024 | 0.6130 |
| Tranfer learning | 0.6963 | 0.6654 |
